# Supplementary material for: Microchannelled alkylated chitosan sponge to treat noncompressible hemorrhages and facilitate wound healing
Source: Nat Commun. 2021 Aug 5;12:4733. doi: 10.1038/s41467-021-24972-2 (PMC8342549; doi:10.1038/s41467-021-24972-2)
Supplement: Supplementary file 1 — Supplementary Information [file 41467_2021_24972_MOESM1_ESM.pdf]

Supplementary information for

Microchannelled alkylated chitosan sponge to treat noncompressible hemorrhages and facilitate wound healing

Xinchen Du[1], Le Wu[1], Hongyu Yan[1], Zhuyan Jiang[2], Shilin Li[1], Wen Li[1], Yanli Bai[1], Hongjun Wang[3], Zhaojun Cheng[4], Deling Kong[1*], Lianyong Wang[1*], Meifeng Zhu[1*]

[1] College of Life Sciences, Key Laboratory of Bioactive Materials (Ministry of Education), Tianjin Center Hospital of Obstetrics and Gynecology, State Key Laboratory of Medicine Chemical Biology, Nankai University, Tianjin 300071, China

[2] Department of Orthopedics, The Second Hospital of Tianjin Medical University, Tianjin 300071, China

[3] Department of Biomedical Engineering, Stevens Institute of Technology, Hoboken, NJ 07030, USA

[4] Shenzhen Traditional Chinese Medicine Hospital, Shenzhen 518000, China

Correspondence should be addressed to D.K. (email: kongdeling@nankai.edu.cn), L.W. (email: wly@nankai.edu.cn) and M.Z. (email: zhumeifeng2013@163.com).

Supplementary Figures

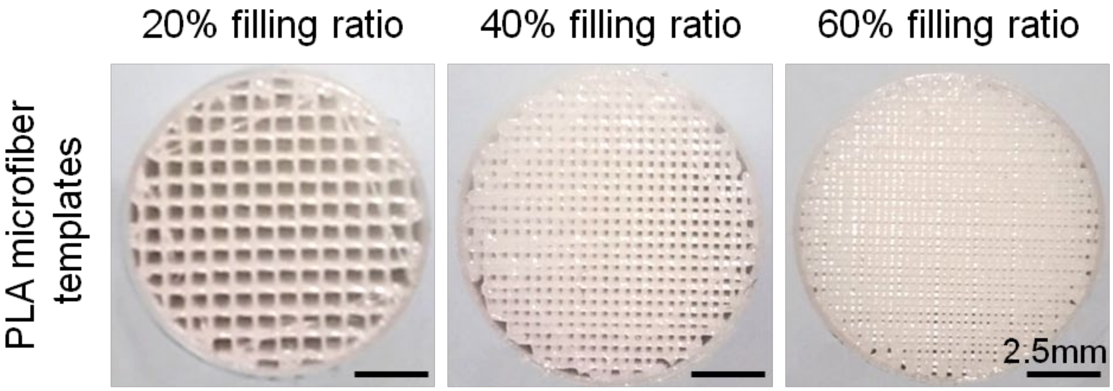

Supplementary Fig. 1 Macro photographs of PLA microfiber templates with filling ratios of 20, 40 and 60%.

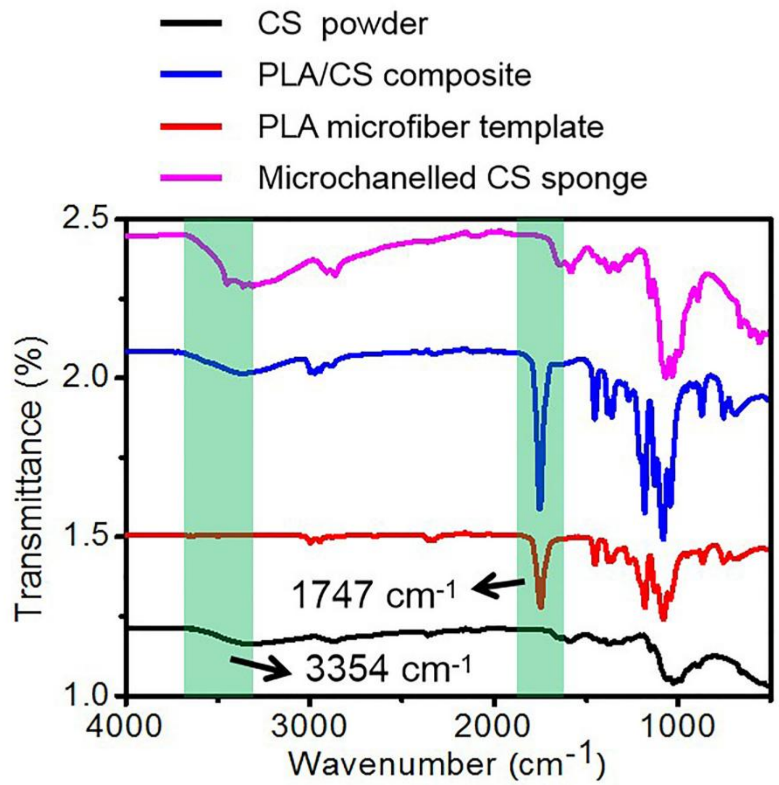

Supplementary Fig. 2 FTIR spectra of the CS powder, PLA microfiber template, PLA/CS composite, and microchannelled CS sponge. In the spectrum of CS powder, the strong peak at 3354cm^{-1} was attributed to the stretching vibration of -NH_2. In the spectrum of the PLA microfiber template, the strong peak at 1747cm^{-1} was ascribed to the stretching vibration of -O-C=O-. In the spectrum of PLA/CS composite, two absorption peaks at 3354cm^{-1} and 1747cm^{-1} were observed, indicating the composition of the CS and PLA. In the spectrum of microchannelled CS sponge, no absorption peak of -O-C=O- was sighted, revealing no residue of PLA microfiber in the sponge.

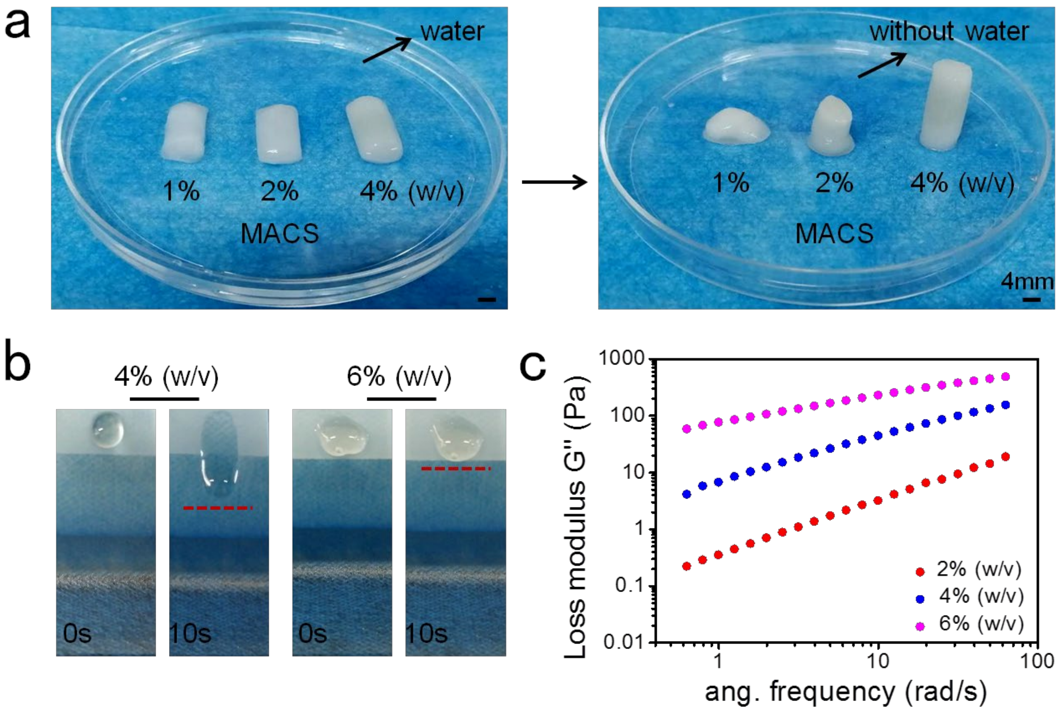

Supplementary Fig. 3 (a) Photographs of the MACS with CS concentrations of 1, 2, and 4% (w/v) in water and dry environment. (b) Photographs of the position of both 4 and 6% (w/v) CS solutions on sloping glass surface within 10s. (c) Rheological property of 2, 4, and 6% (w/v) CS solutions.

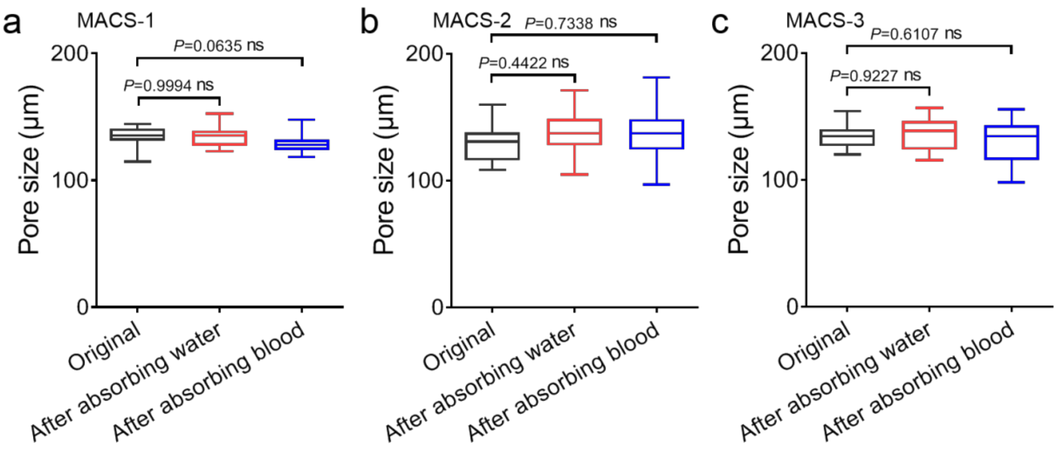

Supplementary Fig. 4 (a-c) Statistical data of pore size of the MACS-1, MACS-2 and MACS-3 before and after absorbing water and blood (pore size, n=16). Data are expressed as mean ± SD. Significant difference was detected by one-way ANOVA with Tukey's multiple comparisons test. The 'ns' indicated no significant difference.

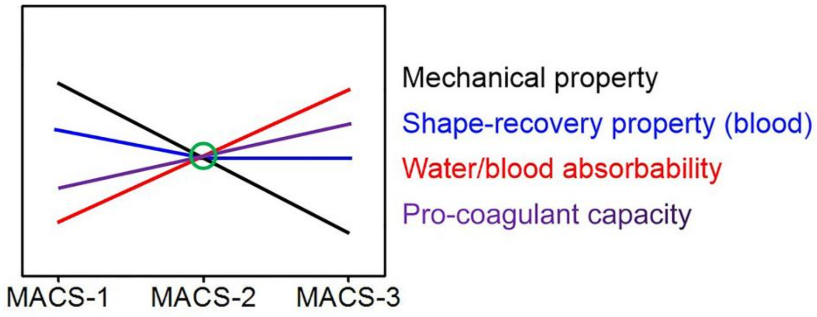

Supplementary Fig. 5 The reason for selecting and using the MACS-2 to conduct in vivo hemostasis, anti-infection, and in situ tissue regeneration experiments.

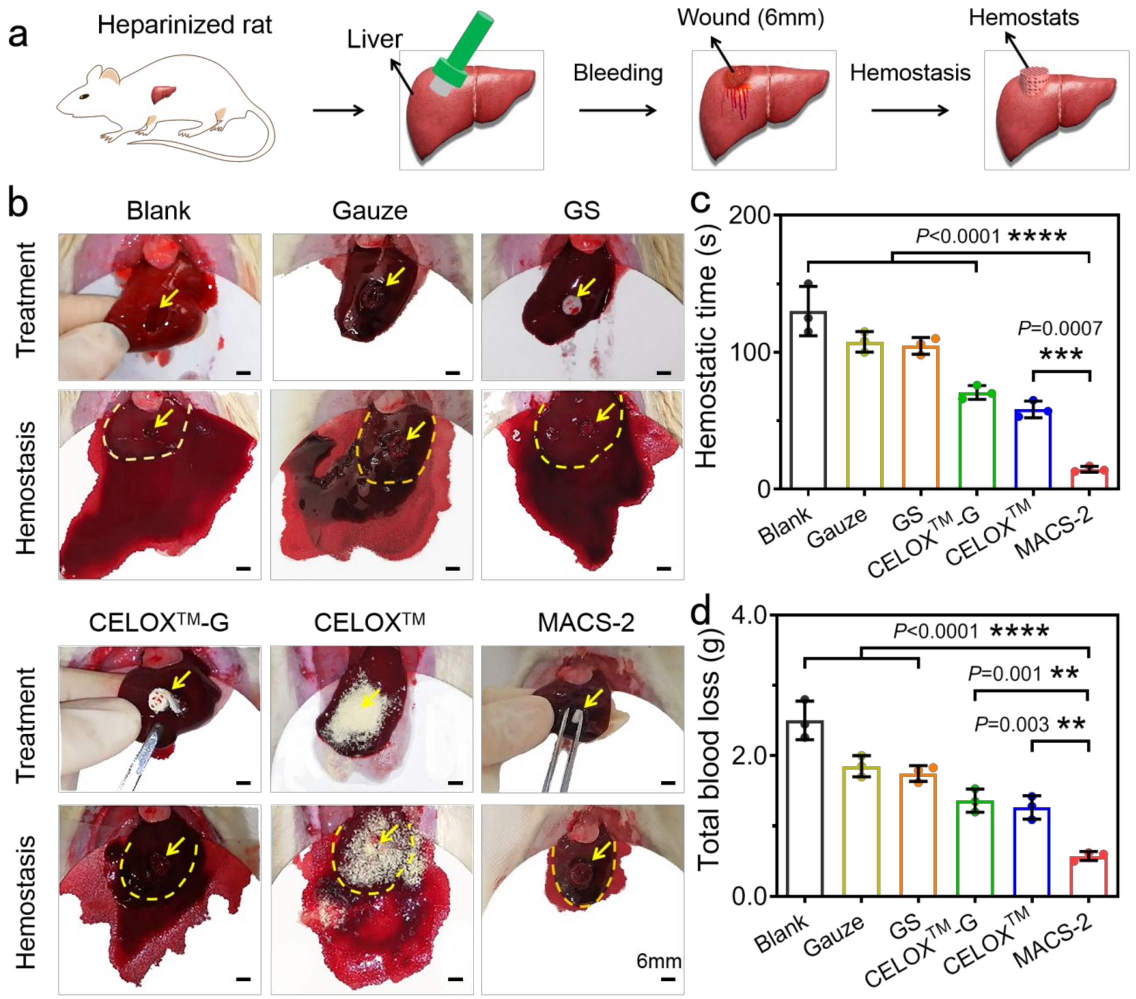

Supplementary Fig. 6 Hemostasis of the gauze, GS, CELOX^{TM}-G, CELOX^{TM}, and MACS-2 in a heparinized rat liver perforation wound model. (a) Schematic illustration of the hemostatic process of hemostats in a heparinized rat liver perforation wound model. (b) Photographs of hemostatic effect of various samples. Yellow arrow and dotted line represented the bleeding site and liver boundary, respectively. (c, d) Hemostatic time and total blood loss in various groups. n=3 rats per group. Data are expressed as mean ± SD. Significant difference was detected by one-way ANOVA with Tukey's multiple comparisons test. **P<0.01, ***P<0.001, ****P<0.0001.

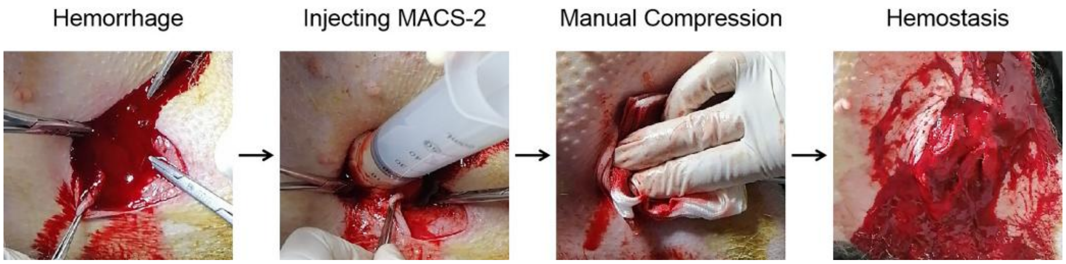

Supplementary Fig. 7 Macro photographs of hemostasis process of the MACS-2 in pig femoral artery bleeding model.

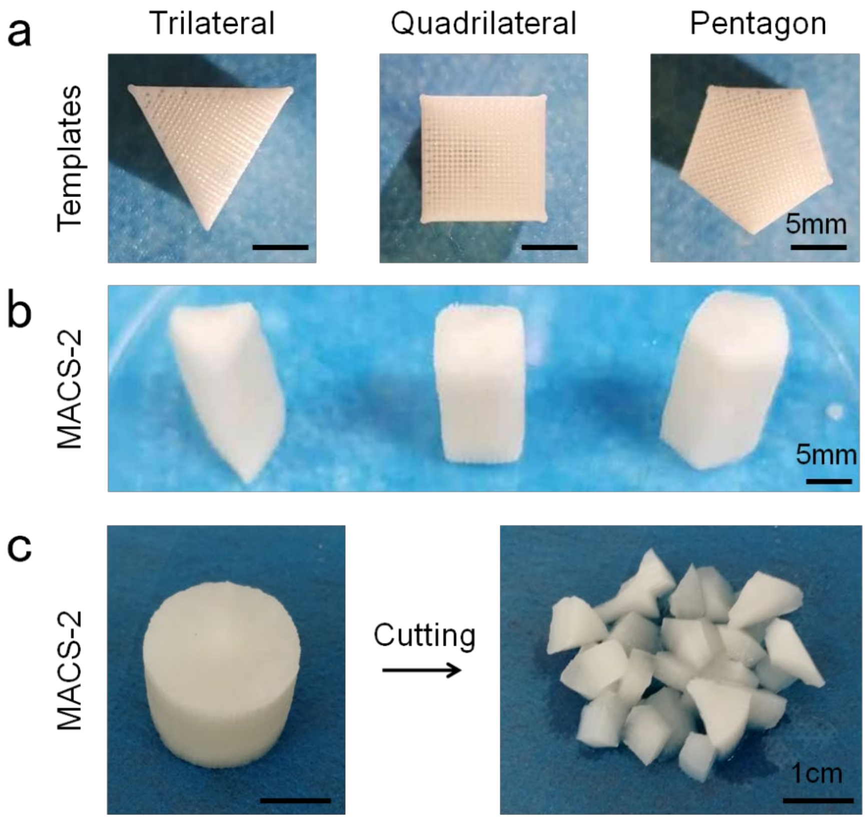

Supplementary Fig. 8 (a, b, c) Macro photographs of PLA microfiber templates, MACS-2 with different shapes, and small pieces of MACS-2.

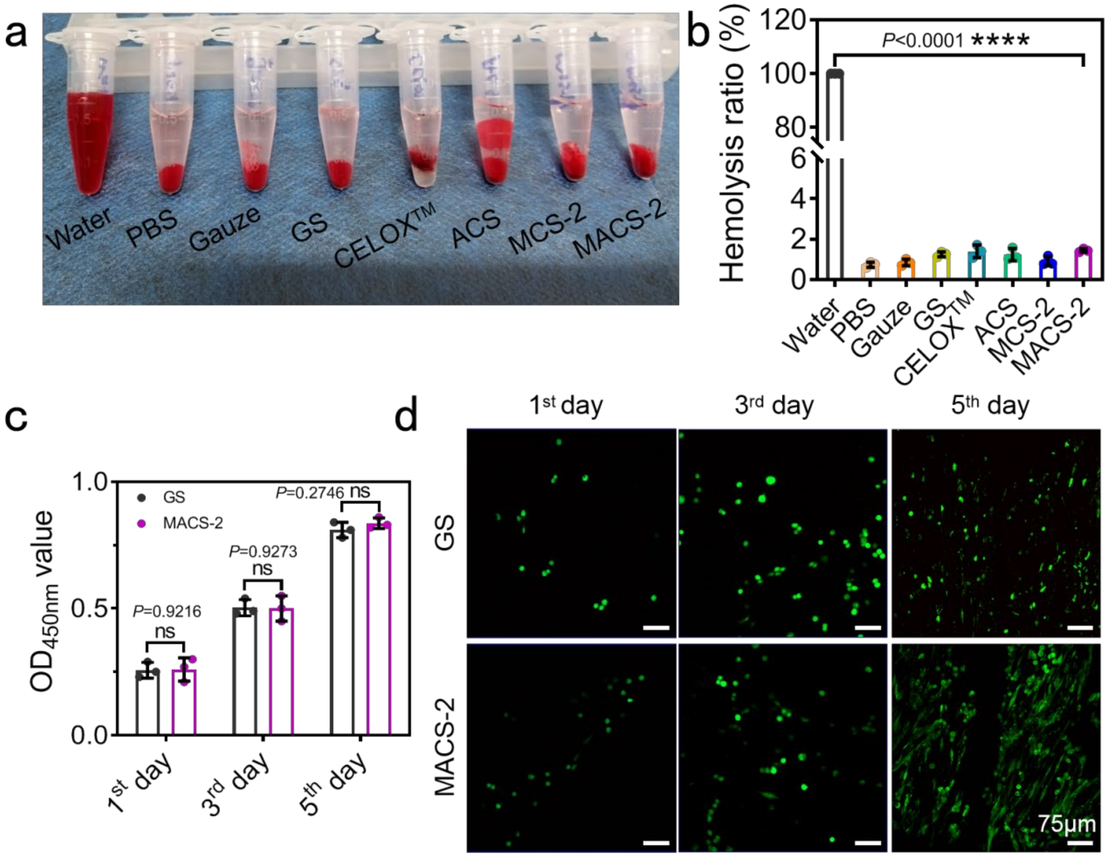

Supplementary Fig. 9 Hemocompatibility and cytocompatibility of the MACS-2. (a) Macro photographs from hemolytic activity assay of water, PBS, gauze, GS, CELOX™, ACS, MCS-2, and MACS-2. (b) Hemolysis ratio in different groups. n=3 independent samples. Data are expressed as mean ± SD. Significant difference was detected by one-way ANOVA with Tukey's multiple comparisons test. ****P<0.0001. (c) The OD_{450nm} value in both GS and MACS-2 groups during 5-day culture. n=3 independent samples. Data are expressed as mean ± SD. Significant difference was detected by unpaired two-tailed t-test. The 'ns' indicated no significant difference. (d) Representative Live/Dead staining images of 3T3 fibroblast cells after culture on the GS and MACS-2 for 1, 3, and 5 days. Each experiment was repeated three times and similar results were acquired.
