# Supplementary material for: Microchannelled alkylated chitosan sponge to treat noncompressible hemorrhages and facilitate wound healing
Source: Nat Commun. 2021 Aug 5;12:4733. doi: 10.1038/s41467-021-24972-2 (PMC8342549; doi:10.1038/s41467-021-24972-2)
Supplement: Supplementary file 3 — Description of Additional Supplementary Files [file 41467_2021_24972_MOESM3_ESM.docx]

Description of Additional Supplementary Files

Title: Supplementary Movie 1

Description: Video of microCT dynamic scanning of the MACS-1.

Title: Supplementary Movie 2

Description: Video of microCT dynamic scanning of the MACS-2.

Title: Supplementary Movie 3

Description: Video of microCT dynamic scanning of the MACS-3.

Title: Supplementary Movie 4

Description: Video of microCT dynamic scanning of the ACS.

Title: Supplementary Movie 5

Description: Video of water-triggered shape recovery of the MACS-2.

Title: Supplementary Movie 6

Description: Video of water-triggered shape recovery of the ACS.

Title: Supplementary Movie 7

Description: Video of blood-triggered shape recovery of the MACS-2.

Title: Supplementary Movie 8

Description: Video of blood-triggered shape recovery of the ACS.

Title: Supplementary Movie 9

Description: Video of hemostasis of the MACS-2 in normal rat liver perforation wound model.

Title: Supplementary Movie 10

Description: Video of hemostasis of the MACS-2 in heparinized rat liver perforation wound model.

Title: Supplementary Movie 11

Description: Video of hemostasis of the MACS-2 in lethal pig liver perforation wound model.

Title: Supplementary Movie 12

Description: Video of hemostasis of the blank group in lethal pig liver perforation wound model.

Title: Supplementary Movie 13

Description: Video of hemostasis of the CELOXTM in lethal pig liver perforation wound model.

Title: Supplementary Movie 14

Description: Video of hemostasis of the MACS-2 in lethal pig femoral artery hemorrhage model.
