# Supplementary material for: Microchannelled alkylated chitosan sponge to treat noncompressible hemorrhages and facilitate wound healing
Source: Nat Commun. 2021 Aug 5;12:4733. doi: 10.1038/s41467-021-24972-2 (PMC8342549; doi:10.1038/s41467-021-24972-2)
Supplement: Supplementary file 18 — Reporting Summary [file 41467_2021_24972_MOESM18_ESM.pdf]

nature research

Corresponding author(s): Meifeng Zhu

Last updated by author(s): Jun 23, 2021

Reporting Summary

Nature Research wishes to improve the reproducibility of the work that we publish. This form provides structure for consistency and transparency in reporting. For further information on Nature Research policies, see our Editorial Policies and the Editorial Policy Checklist.

Statistics

For all statistical analyses, confirm that the following items are present in the figure legend, table legend, main text, or Methods section.

n/a	Confirmed	
☐	☒	The exact sample size (n) for each experimental group/condition, given as a discrete number and unit of measurement
☐	☒	A statement on whether measurements were taken from distinct samples or whether the same sample was measured repeatedly
☐	☒	The statistical test(s) used AND whether they are one- or two-sided<br>*Only common tests should be described solely by name; describe more complex techniques in the Methods section.*
☐	☒	A description of all covariates tested
☐	☒	A description of any assumptions or corrections, such as tests of normality and adjustment for multiple comparisons
☐	☒	A full description of the statistical parameters including central tendency (e.g. means) or other basic estimates (e.g. regression coefficient) AND variation (e.g. standard deviation) or associated estimates of uncertainty (e.g. confidence intervals)
☐	☒	For null hypothesis testing, the test statistic (e.g. F, t, r) with confidence intervals, effect sizes, degrees of freedom and P value noted<br>*Give P values as exact values whenever suitable.*
☒	☐	For Bayesian analysis, information on the choice of priors and Markov chain Monte Carlo settings
☒	☐	For hierarchical and complex designs, identification of the appropriate level for tests and full reporting of outcomes
☒	☐	Estimates of effect sizes (e.g. Cohen's d, Pearson's r), indicating how they were calculated

Our web collection on statistics for biologists contains articles on many of the points above.

Software and code

Policy information about availability of computer code

Data collection	Fourier transform infrared spectrometer (FTIR, TENSOR II, Germany), X-ray photoelectron spectrometer (XPS, Axis Ultra DLD, England), CasaXPS software (Version: 2.3.14), Bruker SkyScan Micro-CT (SkyScan 1276, Allentown, PA, USA), Scanning electron microscopy (SEM, Phenom Pro, Netherlands), Image-J software (Version: 1.44p), Universal mechanical tester (Instron 3345), Software Solidworks Flow Simulation software (Solidworks premium 2016×64 edition, SolidWorks Corp., MA, USA) , Microplate reader (BIO-RAD, iMARKTM), Laser confocal scanning microscope (Leica, Germany), Upright microscope (Leica DM3000, Germany) , Fluorescence microscope (Zeiss Axio Imager Z1, Germany).
Data analysis	FTIR was used to characteristic the chemical groups of samples. XPS was used to characteristic the superficial chemical structure and element content of samples. XPS peak of N1s was treated with the CasaXPS software. Macrostructure and porosity of samples were characterized by the Bruker SkyScan Micro-CT. Microstructure of samples was observed by SEM. Pore size of samples was measured using Image-J software. Mechanical property of samples was evaluated by a compression test using universal mechanical tester. Fluid absorption behavior of samples was assessed via a Software Solidworks Flow Simulation software. The optical value of fluid was tested using a microplate reader. H&E images were acquired by the upright microscope. Fluorescence images were obtained by using the fluorescence microscope and laser confocal scanning microscope. All statistical data were analyzed using Prism 8 (GraphPad) software.

For manuscripts utilizing custom algorithms or software that are central to the research but not yet described in published literature, software must be made available to editors and reviewers. We strongly encourage code deposition in a community repository (e.g. GitHub). See the Nature Research guidelines for submitting code & software for further information.

Data

Policy information about availability of data

All manuscripts must include a data availability statement. This statement should provide the following information, where applicable:

- Accession codes, unique identifiers, or web links for publicly available datasets
- A list of figures that have associated raw data
- A description of any restrictions on data availability

The source data underlying Figs. 1e-n, 2b-d, 3b-g, k, 4c-f, 5a-c, 6c-d, 7c-d, 8c-d, 9b-e and Supplementary Figs. 2, 3c, 4a-c, 6c-d, 9b-c are provided as a Source Data file, and the datasets that support the findings of this study are available from the corresponding author upon reasonable request.

Field-specific reporting

Please select the one below that is the best fit for your research. If you are not sure, read the appropriate sections before making your selection.

[x] Life sciences [ ] Behavioural & social sciences [ ] Ecological, evolutionary & environmental sciences

For a reference copy of the document with all sections, see nature.com/documents/nr-reporting-summary-flat.pdf

Life sciences study design

All studies must disclose on these points even when the disclosure is negative.

Sample size	No statistical methods were used to predetermine the sample size. For all these experiments, three independent repeats/animals were deployed in each group, which allowed sufficient statistics to perform unpaired student t-test or ANOVA analysis, and gave p values to indicate the significance.
Data exclusions	No data was excluded from the analyses.
Replication	All experiments were repeated from at least three independent tests, and all attempts at replication were successful.
Randomization	All experiments were performed randomly in our study.
Blinding	For all experiments, data acquisition and analysis were performed by investigators who are blinded to the groups.

Reporting for specific materials, systems and methods

We require information from authors about some types of materials, experimental systems and methods used in many studies. Here, indicate whether each material, system or method listed is relevant to your study. If you are not sure if a list item applies to your research, read the appropriate section before selecting a response.

Materials & experimental systems

n/a	Involved in the study	
[ ]	[x]	Antibodies
[x]	[ ]	Eukaryotic cell lines
[x]	[ ]	Palaeontology and archaeology
[ ]	[x]	Animals and other organisms
[x]	[ ]	Human research participants
[x]	[ ]	Clinical data
[x]	[ ]	Dual use research of concern

Methods

n/a	Involved in the study	
[x]	[ ]	ChIP-seq
[x]	[ ]	Flow cytometry
[x]	[ ]	MRI-based neuroimaging

Antibodies

Antibodies used	Goat anti-mouse IgG (H+L) (Alexa Fluor 594 Invitrogen™), Thermo Fisher Scientific, Cat# A11032. Dilution: 1:200;<br>Mouse monoclonal anti-ALB (F-10), Santa Cruz Biotechnology, Cat# sc-271605. Dilution: 1:100;<br>Rabbit polyclonal anti- Von Willebrand Factor,Abcam, Cat#ab6994. Dilution: 1:100;<br>Goat anti-rabbit IgG (H+L) (Alexa Fluor 594), Abcam, Cat#ab150080. Dilution: 1:200;<br>Mouse monoclonal [k9218] to HNF-4alpha, Abcam, Cat# ab41898. Dilution: 1:100;<br>Mouse monoclonal anti-P-Selectin (CTB201), Scbt, Cat# sc-8419. Dilution: 1:100.
Validation	https://www.thermofisher.com/cn/zh/antibody/product/Goat-anti-Mouse-IgG-H-L-Highly-Cross-Adsorbed-Secondary-Antibody-Polyclonal/A-11032;<br>https://www.scbt.com/p/alb-antibody-f-10?productCanUrl=alb-antibody-f-10&_requestid=1079782;

	https://www.abcam.cn/von-willebrand-factor-antibody-ab6994.html;<br>https://www.abcam.cn/goat-rabbit-igg-hl-alexa-fluor-594-ab150080.html;<br>https://www.abcam.cn/hnf-4-alpha-antibody-k9218-ab41898.html;<br>https://www.scbt.com/p/p-selectin-antibody-ctb201;

Animals and other organisms

Policy information about studies involving animals; ARRIVE guidelines recommended for reporting animal research

Laboratory animals	Sprague Dawley rats (male, 250-300g, 7-8 weeks) were purchased from SPF (Beijing) Biotechnology Co.,Ltd.<br>Bama miniature pigs (male, 15Kg, 3 months) were purchased from Bainong (Tianjin) Laboratory Animal Breeding Technology Co., Ltd.
Wild animals	This study did not use wild animals.
Field-collected samples	This study did not involve field-collected samples.
Ethics oversight	All animal experiments were performed with the approval of the Animal Experimental Ethics Committee of Nankai University.

Note that full information on the approval of the study protocol must also be provided in the manuscript.
